# Complete mitochondrial genome of *Coenomyia ferruginea* (Scopoli). (Diptera, Coenomyiidae)

**DOI:** 10.1080/23802359.2020.1787889

**Published:** 2020-07-09

**Authors:** Shuangmei Ding, Ding Yang

**Affiliations:** College of Plant Protection, China Agriculture University, Beijing, China

**Keywords:** Coenomyiidae, mitochondrial genome, phylogeny

## Abstract

The dipteran family Coenomyiidae was firstly separated from the Xylophagidae by Akrira Nagatomi in 1975. We sequenced and annotated the mitochondrial genome of *Coenomyia ferruginea*, the first representative of genera *Coenomyia* with complete mitochondrial data. This mitogenome is 17,283 bp totally, which consists of 22 transfer RNAs, 13 protein-coding genes, 2 ribosomal RNAs, and 1 non-coding control region. All genes have the conservational arrangement like other published species of brachyceran flies. The nucleotide composition biases toward A and T, the overall A + T% was up to 75.4% of the entire mitogenome. Both Bayesian inference and ML analysis strongly supported the sister relationship between Coenomyiidae and Xylophagidae. Our results also suggested that Xylophagomorpha is the sister group to Stratiomyomorpha.

Coenomyiidae is a small family containing 6 genera and not over 30 species, which are predominantly distributed in Nearctic, Oriental, and Palearctic regions (Yang and Nagatomi 1994). Adults usually occur in old humid forested areas. The larvae live under bark and in decaying wood and are considered to be predaceous upon other insect larvae (Rozkošný 2005).

As a member of the so-called ‘lower Brachycera’, Coenomyiidae has been with an equivocal identity in the past. Oldroyd (1969) mentioned that ‘…, a number of primitive genera, in various regions of the world, cannot satisfactorily be allocated to any of the larger families. The simplest solution is to assemble all these genera into one family, Coenomyiidae, while recognizing that this is not necessarily a natural unit’. The clear definition of Coenomyiidae was proposed by Nagatomi (1975), who also revised the affiliations of many genera related to this family. However, there is no general agreement about the systematic placement of Coenomyiidae. This family was supposed to be either closer to the Rhagionidae and Tabanidae, or to the Stratiomyidae (Nagatomi 1975). Considering of the fewer members of Coenomyiidae, as well as the assumed close relationship between Coenomyiidae and Xylophagidae, most of the studies involved the phylogeny of lower Brachycera only included the Xylophagidae and neglected the Coenomyiidae (Yeates 2002; Wiegmann et al. 2011; Lambkin et al. 2013; Shin et al. 2018).

We found one published mitochondrial genome of genus *Dialysis* that was used to represent the Xylophagidae instead of Coenomyiidae (Wang et al. 2016), so we provide another complete mitochondrial genome of *Coenomyia ferruginea* from family Coenomyiidae for further multiple phylogenetic analysis. Specimens were collected at Wrightwood, Lone Pine Canyon, CA, USA, the DNA and specimens are deposited in the Entomological Museum of China Agricultural University. The complete data has been submitted to NCBI database with the accession number MT449448.

The sequencing followed the procedures of Gillett et al. (2014), the pooled dsDNA sample was sent to Bionona Co., Ltd, for library construction and sequenced by the Illumina HiSeq2500 platform. The final filtered reads were assembled with Meta-IDBA (Peng et al. 2012).

The complete mitochondrial genome of *C. ferruginea* is 17,283 bp in length and consist of 37 canonical mitochondrial genes and one non-coding control region. The overall nucleotide composition of this mitochondrial genome was 41.4% of A, 34.0% of T, 15.1% of C, and 9.5% of G. The nucleotide composition of control region showed high bias toward A and T which the A + T% is up to 85.9%.

The ATG was the most popular start codon shared with ATP6, CO2, CO3, CYTB, ND4, ND4L, and start codon ATT was shared with ATP8, ND2, ND3, ND5, ND6. Particularly, the CO1 begins with codon CTG, and the ND1 begins with codon ATA. The conservative stop codon TAA was shared with most of the PCGs except for three genes, CYTB and ND3 were terminated with stop codon TAG, while the gene ND5 was ended with an incomplete stop codon T.

Phylogenetic trees were inferred using two approaches BI on MrBayes v3.2.6 (Ronquist et al. 2012), and ML on RAxML-HPC2 v8.2.10 (Stamatakis 2006) for dataset containing 13 PCGs and 2 ribosomal RNA genes (Figure 1). *Tipula abdominalis* was chosen as an outgroup. The monophyly of the Xylophagomorpha and Stratiomyomorpha were consistently supported, which is the same as the recent mitochondrial (Wang et al. 2016) and morphological (Lambkin et al. 2013) studies. The sister-relationship between Xylophagidae and Coenomyiidae provides new phylogenetic evidence based on mitochondrial genome data: despite the divergence of adult morphology and larval feeding habits, the Coenomyiinae should be united into the infraorder Xylophagomorpha, and the Xylophagomorpha are nearer to the Stratiomyomorpha (Nagatomi 1975).

**Figure 1.**
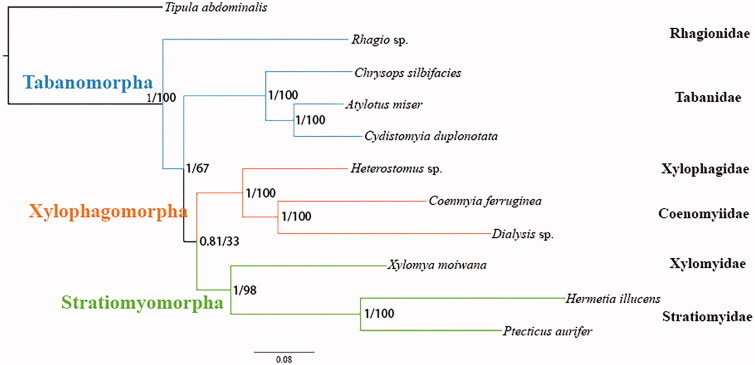
Phylogenetic tree of Brachyceran species which consist of two Coenomyiidae species. Bayesian posterior probabilities and ML bootstrap values were labeled at each node. The GenBank accession numbers for all species: *Tipula abdominalis* (JN861743.1) ; *Rhagio sp*. (KT225298.1); *Chrysops silvifacies* (KT225292.1); *Atylotus miser* (NC030000.1); *Cydistomyia duplonotata* (NC_008756.1); *Dialysis sp*. (KT225293.1); *Heferstomus sp*. (MH817480); *Coenomyia ferruginea* (MT449448); *Xylomya moiwana* (KT225302); *Hermetia illucens* (NC_035232.1); *Ptecticus aurifer *(MN604259.1).

## Data Availability

The data that support the findings of this study are available in NCBI at http://www.ncbi.nlm.nih.gov/, the reference number is MT449448.
